# Unregulated care providers’ engagement in palliative care to older clients and their families in the home setting: a mixed methods study

**DOI:** 10.1186/s12904-019-0442-5

**Published:** 2019-07-06

**Authors:** Christine J. McPherson, Judy Etele, Viviane Chou-Yin Ta, Angelina Raghubir

**Affiliations:** 1 School of Nursing, Faculty of Health Sciences, University of Ottawa, Guindon Hall, (3045) 451, Smyth Road, Ottawa, Ontario K1H 8M5 Canada; 2 School of Psychology, Faculty of Social Sciences, University of Ottawa, 136 Jean-Jacques Lussier, Vanier Hall, Ottawa, Ontario K1N 6N5 Canada

**Keywords:** Client care, Palliative care, Aging, Unregulated care provider, Home care worker, Support worker, Home care, Family caregiver, Mixed methods

## Abstract

**Background:**

Unregulated care providers (UCPs) are at the forefront of direct client care in the community. Their services are required to meet the demand for home-based palliative care from a growing older population, yet understanding of UCPs involvement in care is limited. The study aimed to identify the types and frequencies of tasks performed by UCPs in home-based palliative care to older clients (> 65 years) and their families and to describe UCPs’ engagement in care, and barriers and facilitators to their work.

**Methods:**

A mixed method approach was used comprising a quantitative retrospective chart review of UCPs’ tasks (*n* = 66), qualitative content analysis of progress notes from clients’ charts (*n* = 85), and thematic analyses of in-depth interviews with UCPs (*n* = 10).

**Results:**

A thematic structure was derived from analyses and integration of data from the chart review and interviews. The themes reflect the physical, affective, and relational aspects of UCPs involvement in the care of clients and families at the end of life. The findings indicate that although a significant proportion (63%) of the 13, 558 UCP tasks identified were directed toward meeting clients’ physical care needs, their presence in the home, made UCPs an important source of information on the client’s condition; observing and appraising the situation. Further, the nature of their work and frequent interactions with clients and families also presented opportunities for UCPs to provide emotional support; a role UCPs felt was integral to their work.

**Conclusions:**

The study highlights the challenging nature of palliative care to older clients and their families whose needs are often complicated, situated within the unique environment of home care where supervision of UCPs is at a distance. Challenges and facilitators to UCPs’ work in this context are discussed with recommendations to support UCPs in their roles.

## Background

Access to palliative care is highly variable and significant numbers of people do not receive it [1]. People who are older (65 years and over) and those with non-cancer progressive illnesses (e.g., cardiac, pulmonary, renal, neurodegenerative, and dementia) and frailty- most prevalent in advanced age- are less likely to receive palliative care [2]. There is an emphasis on the integrating palliative care into the continuum of care and training of non-specialist (generalists) healthcare providers to deliver palliative care to improve access; making it available outside of specialized settings (i.e., hospice and palliative care units) into long-term care and home care [1]. At the same time, an emphasis on aging-in-place and preference by people with a life-limiting illness to remain at home has intensified the demand for home-based palliative care [3]. Palliative care in the home is overseen by healthcare professionals (HCPs) including physicians, nurses, physiotherapists, occupational therapists, and social workers, as part of a multidisciplinary team approach to care. However, HCPs contact with home care clients and families are increasingly limited due to human resource and financial constraints. Instead, there is reliance on family caregivers (FCGs), and unregulated care providers (UCPs) to provide care to the client on a day-to-day basis [4, 5]. The higher likelihood of multi-morbidity, polypharmacy, and age-related functional declines in older clients can add to the level of complexity in palliative care making it crucial that FCGs and UCPs receive appropriate training and support. There is widespread recognition of the significant role FCGs’ play in the provision of palliative care in the home [5]; however, much less is known about the roles and responsibilities of UCPs in shaping the palliative care experiences of older clients and their families.

Estimates indicate that UCPs provide approximately 80% of direct care to Canadians 65 years and over in the community [6]. UCPs comprise a significant proportion of health care providers within the Canadian health care system, and their numbers are expected to increase as the demand for homecare grows with the aging of the population; a pattern observed in other developed countries [7, 8]. UCP is one of many titles assigned to health care workers that provide services to meet the health and social needs of clients across settings (hospital, long-term care, and home), and are not licenced or regulated by a regulatory or professional body [4, 9]. Amongst others, titles include personal support workers, care workers, home support workers, home care workers, personal carers, nursing aides, and nursing care assistants [8].

There is recognition that UCPs have a vital role in palliative care as part of the health care team [10, 11]. UCPs responsibilities typically include assisting clients with activities of daily living (e.g., personal care), and practical support (e.g., shopping and housework), and providing respite for FCGs [10, 12, 13]. In delivering this care, UCPs also provide psychosocial support [10, 13]. Growing demand for home care services and fiscal pressures has resulted in UCPs’ roles evolve from a supportive one to encompass responsibilities (e.g., wound care, exercise and range of motion exercises, bowel and ostomy care, assistance with medications, blood glucose monitoring, and tube feeding), referred to as *controlled acts,* which are conducted by HCPs, and are delegated by a HCP [14]. However, UCPs do not have a professional designation or accredited training and education; therefore, their competencies and scope of practice are not clearly defined [4, 8]. Consequently, there is considerable variability in UCPs’ knowledge, preparation, and responsibilities [4, 7]. UCPs may receive little (if any) palliative care training, and research indicates that they may be ill-prepared to care for clients at the end of life and lack knowledge and comfort dealing with death and dying [13, 15].

An understanding of UCPs involvement in palliative care is essential for preparing and supporting UCPs in their role and for the provision of high-quality palliative care. However, research is lacking. Herber and Johnson, in a review of the literature examining UCPs’ roles in palliative care to older clients, identified eight studies [10]. However, the majority of the studies in their review focused on UCPs in residential and long-term care rather than the home [10]. Though there are likely to be similarities between UCPs working in inpatient settings and clients’ homes, there are also some important distinctions. One crucial distinction is that UCPs in inpatient settings are directly supervised and work alongside other health care providers; thus, they have access to continuous support [16]. In contrast, UCPs in the home tend to work independently, and supervision is at a distance. The home setting is also more unpredictable; UCPs in the home, therefore, have to deal with a highly variable and changing working environment [17]. Further, an examination of the literature on UCPs in home-based palliative care for older clients revealed no literature since Herber and Johnson’s review in 2013.

### Aims

UCPs are at the forefront of direct client care to older clients at the end of life and their families; a role that is evolving to meet the demand for home-based palliative care. Even so, there remains little research aimed at understanding UCPs’ roles and responsibilities, and how they engage with older clients and their families to shape their palliative care experiences. This study aims to: identify and describe the types and frequencies of tasks performed by UCPs in the provision of home-based palliative care to older clients and their families, describe UCPs’ engagement in home-based palliative care, and identify barriers and facilitators to their work.

## Methods

### Design

An exploratory two-phase sequential mixed method design was employed.

#### Phase 1

The types and frequencies of UCPs’ tasks were investigated with a quantitative descriptive design, using a retrospective chart review methodology. Qualitative data was also collected from clients’ charts, and content analyzed to add to an understanding of UCPs’ roles and responsibilities, and engagement with clients and families.

#### Phase 2

To describe UCPs’ engagement in palliative care and identify barriers and facilitators to their work, we used a qualitative descriptive approach that incorporated relational and contextual aspects of UCPs’ subjective experiences. An inductive thematic analysis was used to analyze data collected from in-depth semi-structured interviews with UCPs. Thematic analyses as Braun and King point out are not bound to any theoretical framework and can be used within different theoretical frameworks [18]. The approach is commensurate with our pragmatic stance.

### Setting and recruitment

Research ethics approval was granted from the university where the investigators are affiliated, and approval from the recruiting organizations. Recruitment was through the Palliative Care Program of one Local Health Integration Network (LHIN) that coordinates, refers, and provides community health care services, and three home care nursing agencies in Ontario, Canada. The recruiting organization provides homecare services to almost 9,000 clients; of which 1200 are within the Palliative Care Program.

### Phase 1: retrospective chart review

#### Chart eligibility and sampling

The Chart-in-the-Home (CITH) is a vital source of information on client care and includes care plans, client risk assessments, medications, UCP task list, and progress notes. A consecutive sampling approach was employed including the CITH of clients (> 65 years), who were discharged from the LHIN in the previous 12 months and had received palliative care services in the 3 months before discharge (clients’ death or transfer to inpatient services). For the sampled charts to be included, the clients must have received UCPs services in the 3 months before discharge; criteria determined through examination of the CITH. Of the 87 charts examined, 21 were excluded as the client had received little (less than three) if any UCP visits.

Table 1 contains the characteristics of the sample from the chart review. Given the consecutive sampling approach, 1666 UCP visits examined in the charts, and numbers of UCPs employed within the Palliative Care Program (> 500), we estimate that the charts included a sample of at least 200 UCPs.

**Table 1:** Descriptive statistics of client characteristics from the retrospective chart review

	Clients (n = 66)
Age (mean, SD)	80 years (8.89)
Age (range)	66–98 years
Sex	
Male	31 (47%)
Female	35 (53%)
Primary diagnosis	
Cancer	44 (66.7%)
Non-cancer	22 (33.3%)
Secondary diagnosis (e.g., arthritis, dementia, heart disease, diabetes)	
None	21 (31.8%)
1–2	40 (60.6%)
> 2	5 (7.6%)
Died at home	40 (60.6%)
Discharged	26 (39.4%)
Family caregiver	63 (95.5%)
Partner	30 (45.5%)
Adult child	39 (59.1%)
Extended family/friends	7 (10.1%)

The UCP task list (in the CITH) is the primary source of information on UCPs’ activities. The UCP task list is a structured form, with 13 predetermined headings that correspond to activities of daily living, instrumental activities of daily living (e.g., meal preparation), emotional support, and interventions (e.g., therapeutic activities). The UCP task list is the same for all clients receiving care through the LHIN. At each visit, the UCP records his/her activities by initialing in a box on the UCP task list that corresponds to the tasks to indicate that he/she has completed it. The types of tasks assigned to UCPs vary depending on the clients’ and families’ needs. Although the UCP task list a structured format, there are two headings identified as “other” where UCPs can add to the form. Also, there is a heading identified as “progress note/verbal report” where UCPs can indicate that they have added a progress note or made a verbal report to a supervisor. As the UCP task list is highly structured with limited space to write, “other” and reference to a progress note are typically indicated as more information can be provided in the progress notes. If an assigned task is not completed the UCP records this on the task list and can add a progress note to explain why the task is not completed.

In line with recommended best practices in chart review, we developed a protocol with guidelines detailing the types of data with definitions of tasks and the grouping of task list headings, how the data is abstracted, dealing with missing data, and inclusion/exclusion criteria [19]. All authors were involved in data collection. Each chart was independently reviewed by one of the authors. We discussed any ambiguities, issues of missing data, and modifications to the data abstraction tool collectively.

**Fig. 1:**
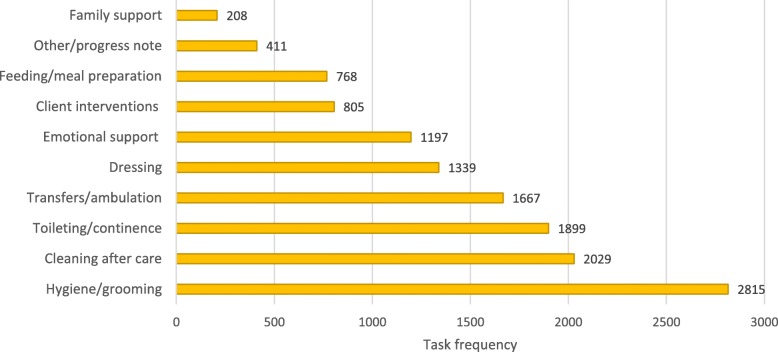
Frequency of UCP task groupings from 1666 home visits in the 3 months before client discharge from home care services. Legend: *Client interventions* combine UCP task list headings: *interventions, therapeutic activities/range of motion; medications (eye/ear drops, oral, puffers, transdermal,* and *bowel enema/suppository)*, and *non-sterile dressing*. *Family support* combines UCP task list headings: *family communications, respite, resources/planning, information sharing,* and *support for grief and loss*

Data was collected from the date of discharge backward up to the previous 3 months or the time at which the client was admitted to the service (if < 3 months). Intercoder reliability was calculated using a random number selection to identify 10% of charts using the unique identifier assigned to each chart, following data cleaning and verification for any errors in data entry. A two-way mixed intraclass correlation coefficient (ICC) was calculated for each of the task groupings, following recommended procedures for assessing the level of agreement between raters (Fig. 1 contains the task groupings) [20]. IBM SPSS Statistics for Windows (Version 24) was used for all quantitative analyses. Across the UCPs’ task groupings, ICCs ranged from .88 (*p* < .0.001) for *emotional support* to .98 (p < .0.001) for *family support;* indicating good to excellent intercoder reliability. Descriptive statistics were calculated to provide frequencies, central tendencies, proportions, and ranges.

#### Qualitative data collection and analyses

In the CITH, “progress notes” are freely written notes detailing UCPs’ activities, observations, and communications. Progress notes are not documented at every UCP visit. Only during a visit when the UCP wants to record pertinent observations (e.g., changes in clients’ status and symptoms) and provide greater detail on his/her activities (e.g., interventions and interactions). Of the 66 clients whose CITH were reviewed, 44 (67%) clients had progress notes from UCPs. In total there were 85 separate UCP entries in the progress notes. Data from the progress notes were entered directly into the data extraction tool. As the data is restricted to what is documented limiting interpretation, the first author (CM) used manifest content analysis to reduce the data into a smaller number of content-related categories [18]. Five main categories were identified: *observing and appraising, information-sharing, family presence and participation, physical care needs,* and *implementing interventions.* A second researcher (VT) independently deductively coded the progress notes using the categories identified. Interrater reliability was tested to examine the consistency between coders. Kappas ranged from *k = .66* (*p* < .0.001) for *observing and appraising* to *k = .97* (p < .0.001) for *family presence and participation* indicating good to excellent agreement.

#### Participant eligibility and sampling

Purposeful sampling was used to recruit UCPs with knowledge and experience caring for older clients with life-limiting illnesses in the home and their families. Information regarding the study was sent to all UCPs in the Palliative Care Program. To be eligible UCPs had to: have cared for at least two clients (> 65 years) receiving palliative care services; have worked for one of the three home care agencies where recruitment occurred for at least 3 months (to permit any in-service training); and, speak one of the official languages (English or French). In total, 14 UCPs who were eligible contacted the the first author (CM) to take part in the study. The sample size was based on informational redundancy, which occurs when no new information or themes are derived from the data collection/analyses. Four UCPs were not interviewed. Of these, two could not be reached for an interview, and two were not interviewed as informational redundancy was reached at ten participants. All participants were female; their ages ranged from 27 to 60 years; the mean age was 47 years. Participants were experienced with between 5 and 26 (mean 16.9) years working as a UCP. While there is some variability in how the home care agencies manage care, all agencies have the chart-in-the-home (CITH), used for the retrospective chart review, and provide in-house training and opportunities for UCPs to obtain additional training outside of the organization. All the UCPs who took part in the interviews had received some level of training in gerontology and palliative care through in-service training and community colleges. The sample was ethnically diverse. More than half of the sample being immigrants to Canada and over a third from visible minorities (details are not provided to protect participants’ anonymity).

#### Data collection and analyses of semi-structured interviews

Written informed consent was obtained from participants before data collection. Interviews were conducted over the telephone by two researchers (JE and AR). At the beginning of the interviews, we collected information from participants to describe the sample (i.e., age, gender, culture/ethnicity, work experience as a UCP). Next, to address our aims, we used open-ended questions exploring participants’ roles and responsibilities, their interactions with clients and families, and barriers and facilitators to their work. For example, *“What is your role in addressing the needs of older clients and their families receiving palliative care?”* Emergent areas were explored during the interviews including preparation for the UCP role, UCPs’ roles as part of the health care team, and UCPs’ thoughts and feelings caring for clients at the end of life and their family members. Interviews were digitally recorded except for one participant who preferred not to be recorded. The digital recordings and interview transcriptions were entered into NVivo 10 (QSR International, 2012) to facilitate the organization of the data for analysis. We recognize that our experiences, knowledge, and assumptions have a bearing on our interpretations. We are reflexive in stating that the research team comprised three Registered Nurses (CM, JE, and AR), with experience caring for older clients that are dying. Two team members (CM and AR) have experience working as UCPs in the community, and two (CM and VT) have training in psychology.

The first author (CM) analyzed all the data, and other members of the research team listened to the interviews; each analyzed at least three different interviews in-depth. Consistent with Braun and King’s approach to thematic analysis, the analysis moved beyond the surface level, to explore the data in a more nuanced way [21]. We analyzed participants’ responses, including their feelings, explanations, and interactions; essentially uncovering the meanings attributed to their experiences to create themes to address the aims. Boyatzis describes themes as “a pattern in the information that at a minimum describes and organizes the possible observations and at maximum interprets aspects of the phenomenon” (p. 161) [22]. Memos were kept to document the analytic process and decisions as an audit trail. A preliminary thematic structure was developed based on our analyses.

### Integration of the data

Integration of data is a crucial component of mixed methods and can occur at various stages of the research process [23]. We used a process of merging to fully integrate the data and our interpretations from phases 1 and 2. Merging involves identifying linkages between the data, and comparing and contrasting the findings for similarities and differences, and making inferences on the data as a whole [23]. The authors came together to discuss the findings from phase 1, their observations, thoughts, and interpretations of the data from phase 2. Following this meeting, modifications were made to assimilate the team’s interpretations and integrate the data. To improve integration rather than presenting the findings separately, the first author (CM) used a process of “weaving” to fully integrate the data into the narrative presented in the findings. Weaving involves “writing both qualitative and quantitative findings together on a theme-by-theme or concept-by-concept basis” (p. 2142) [23]*. All authors reviewed and agreed upon the interpretations and presentation.*

## Results

**Fig. 2:**
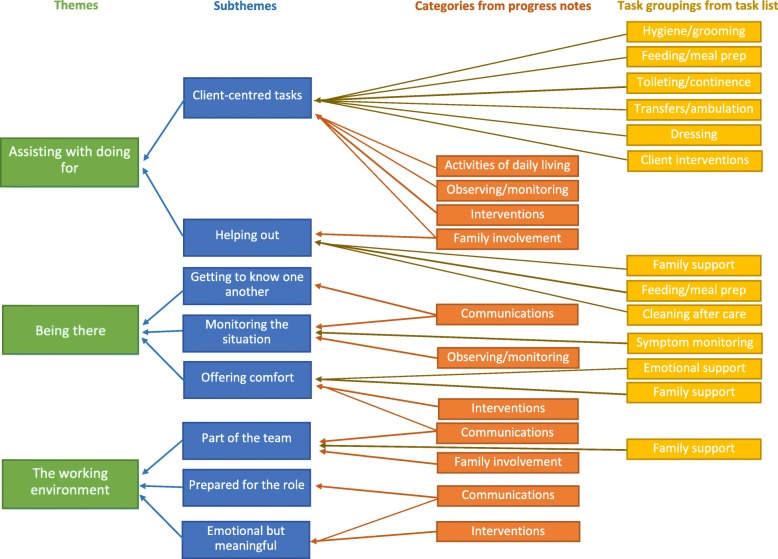
The thematic structure of the findings with links from the progress note categories and UCP task groupings to the subthemes and themes. Legend: *Symptom monitoring* is from the task grouping *other/progress note*

The types and frequencies of UCPs tasks are presented in Fig. 1 and comprise 13,558 instances of tasks carried out during 1666 visits. UCP tasks are presented within the thematic structure, along with categories from a content analysis of 85 UCP progress notes (Fig. 2).

### Assisting with and doing for

The theme *assisting with and doing for* represents the types and level of physical tasks performed by UCPs. Participants characterized their functions in terms of tasks usually prefixed with the level of support depending on the clients’ needs from promoting and helping the client to perform a task (*“assisting with”*), on a continuum to dependence (*“doing for”*). The subtheme *“Engaging in physical care”* reflects the strong focus on clients and their physical needs; while, *“helping out”* comprises other tasks performed by UCPs that supported clients’ and their families.

#### Engaging in physical care

Analysis of UCPs tasks revealed that activities of daily living accounted for 8488 (63%) of activities (Fig. 1). While tasks were directed at clients’ physical needs, UCPs viewed these activities intrinsic to client-centred palliative care. These tasks were a way of engaging with clients; to convey caring and respect, as the following UCP describes.

*“It will depend – I could be doing meal preparation, from bathing to dressing, personal care that kind of stuff. I have one woman who is in a wheelchair; she is palliative. I wash and curl her hair. I do anything that has to do with personal care, doing her fingernails, making her feel good. Reminding them how to feel alive; that they are not forgotten.” (UCP 6)*

UCPs were mindful of the limited time clients had to live and the importance of respecting their wishes. The category *physical care needs* from the progress notes provided detail on what was done for clients, including their preferences and refusal of care. There were 100 references in the progress notes to clients’ preferences regarding care. UCPs were willing to forgo some tasks allocated:

*“My job is to make their day a little bit better. Help with the showering, feeding, depending on the client. Nothing is in stone. If the client is having a bad day and doesn’t want to shower, but just wants you to listen. If that is what makes them feel better, then that is what I do.” (UCP2)*

In providing for clients’ physical needs, UCPs were responsible for administering interventions. *Client interventions* were charted in 805 (5.9%) instances on the UCP task list and included therapeutic activities/ range of motion; medications, and non-sterile dressing (Fig. 1). The category *implementing interventions* from the progress notes provided more information on the interventions. Sometimes interventions were planned (e.g., apply creams); other times, they were implemented in response to clients’ changing needs. Unplanned interventions, for example, were implemented by UCPs in response to symptoms like pain, which regularly occurred. The category *implementing interventions* revealed that UCPs resorted to non-pharmacological interventions for managing symptoms such as massage, compresses, applying creams, and positioning as they were not authorized to administer pharmacological interventions.

*“I try to put myself in the client’s shoes. Massage if they’re in pain … put on some lotion to make them more comfortable. These extras can bring some comfort. It can be scary when they can’t breathe. You want to do more but you can’t. We are not a nurse or doctor. Their [clients] position is very important, so we help and support. So we bring some pillows, we sit up the person, this can help the person have better breathing, we can also open windows.” (UCP9)*

#### Helping out

UCPs’ responsibilities extended to the family and instrumental activities of daily living. UCPs viewed these tasks as *“helping out.”* Providing respite for family caregivers was seen as *“helping out”* the family by *assisting with and doing for* the client, tasks that the family caregiver typically performed, or was unable to do.

*“The family is grateful. I am there to help them and help with what they can’t do. Most of the families find it very difficult to wash the person because they know they are dying. With us there, we are willing to do it. We are supporting the family. We are supporting the person.” (UCP4).*

Respite meant that FCGs could take a break from caregiving to attend to other responsibilities (e.g., doctor’s appointment). Despite UCPs viewing respite as important part of their role, the charts revealed that respite occurred on only 108 of the 1666 home visits (6%). Beyond respite, UCPs facilitated the care that FCGs provided to the client (identified in the category *family presence and participation* from the progress notes) by offering practical advice, *“Wife giving morphine often, suggested a book to keep track of dose.” (Chart 42).*

Few instrumental activities of daily living were identified (15%) and amounted to meal preparation and the UCP task *clean up aftercare,* which involved cleaning after performing a task for a client such as a bathroom after bathing/showering, changing bedclothes, and work surfaces following meal preparation (Fig. 1). Although housework was not identified on the UCP task list, some participants performed cleaning as a way to help out.

*“A lot of times there is the husband and wife; the wife may not be able to do certain things and as a support, you are there to give her a little helping hand, so we do little light housekeeping, vacuum, sweeping… I do a load of laundry, so she can have her time with herself. Little things like that.” (UCP4)*

### Being there

The chart review revealed that UCPs made 1666 (range 3–164) UCP home visits. As there was a wide range in the frequencies of UCP visits to each client, we calculated the median as a measure of central tendency (*Mdn* = 11). Though there was variability in the number of visits depending on the circumstances and client’s needs. As one participant described:

*“You can have a client ongoing for six months, or a year until they are at that end of life stage. You can have them for a week. It really does fluctuate how far along they are. And you really don’t know. You may go in for one night, overnight, an eight-hour shift, and they might be okay, and they pass during that time.” (UCP3)*

Signatures on the charts indicated continuity in the provision of services from UCPs, and that UCPs were in the home more frequently than other health care providers. The theme *“being there”* represented not just physical presence concerning the amount of UCPs’ physical contact with clients and families, but actively being present in terms of building relationships, monitoring the situation in the home, and providing emotional support by listening, companionship, and reassurance. *“Being there”* is reflected in the subthemes *“Getting to know one another,” “Monitoring the situation,”* and *“Offering comfort.”*

#### Getting to know one another

The subtheme “*getting to know one another”* reflected the relational components of UCPs’ roles and was a critical aspect of care centred on clients’ and families’ needs. Consistency in UCPs’ client assignments was seen as a benefit since participants and clients had an opportunity to establish rapport, and build trusting relationships. In some instances, it permitted participants the chance to become familiar with clients before the client’s health declined and communication was affected.

*“You do form relationships you can’t do the care without a relationship or rapport because they’re at the end of their lives. And sometimes it is your face that they see more than anyone else. And you’re the one that helps them with everything.” (UCP10)*

Participants encountered some challenges that hampered their efforts to develop connections with clients and families. Based on the interviews with UCPs and in the category *information-sharing* from the progress notes, several barriers to communication were identified. The main barrier was the client’s physical and psychological functioning (e.g., cognitive impairment, depression, and weakness), and emotions (e.g., aggression), as identified in the following chart entries:

*“Prepared breakfast, but I had to put a hold on it because of his aggression towards me. Very angry. Client very aggressive, grinding teeth at me, so I stepped away for a few minutes. Client says he needs to smarten up about being mad. I told him I understand.” (Chart 52).*

*“Client lying awake in bed, will not speak. Very depressed. Not sure why she won't speak to me. Had a nice conversation yesterday. I have been here now for 15 minutes and trying to make small talk. Client refuses to speak to me. I have tried everything to get her to speak. Nothing.” (Chart 35)*

Though language was not a common issue, participants did identify some difficulties interacting when the first language of the UCP and client was not one of the official languages (English and French). Further, two participants’ experienced negative attitudes because of their ethnicity, as one participant stated, *“I feel judged by my ethnicity from both client and families at times, but once they get to know me and see that I am helping, it’s okay.” (UCP1).*

#### Monitoring the situation

The frequency of UCPs’ interactions with clients and families facilitated monitoring the situation in the home. *Symptom monitoring* was an expected part of UCPs' roles. However, completion of this task was indicated by a check mark on the UCP task list (grouping "other/progress note" in Fig. 1). The progress notes were used as a place to document the clients’ and families’ physical, social, and psychological status. These observations were captured in the category *observing and appraising*, where there were numerous entries (*n* = 420). *Observing and appraising* included notes on clients’ oral intake, urine output, and elimination, as well, as skin integrity, and other pertinent observations (e.g., sores, abrasions, and symptoms). Based on their observations, UCPs had to appraise the situation to determine what was important to record and whether and what, if any, action was required. Care of clients was complicated as over half of the clients had co-morbidity (Table 1). Consequently, clients’ conditions fluctuated, and with declining health, changes in clients’ status occurred more often; necessitating ongoing appraisal by the UCP. Continuity in client care was seen to facilitate monitoring for changes in clients’ status. “*Client in bed resting when I arrived. Shortness of breath better than last week. Yet comfortable.” (Chart 25).*

Although the focus of UCPs' observations was mainly on clients’ physical condition, they occasionally referred to clients’ mental status and emotions, and concerns regarding safety.

*“Client has no energy, and has a lot of pain in the right side of chest and shoulder, weak in legs, confused, cannot follow the conversation and has no strength.” (Chart 42)*

There were fewer references to *observing and appraising* families. Only two progress notes mentioned FCGs ability to cope. ***“****Wife is very tired. Wife up too many times in the night to reposition husband. Exhausted. Husband shouting through the night”. (Chart 26).*

#### Providing comfort

Participants frequently referred to providing comfort to the client and family. Comforting was described in relation to emotional support by listening to clients’ and families’ concerns, offering reassurance, and doing what they could to make “*life a little easier.”* As relationships developed, clients and families were more open to discussing emotional and personal issues:

*“A lot of it is supporting within the home. Also, as a companion as well. Confidant at times. They [clients and families] open up quite often to you on a personal level. So, that is the personal care that we do in the home.” (UCP3)*

The importance of emotionally supporting the client was strengthened by analyses of the charts, which revealed 1197 references to *emotional support* (Fig. 1). As a proportion of all completed tasks, *emotional support* accounted for 11.3% and was recorded at 71.8% of visits. Of the 66 charts reviewed, 52 (78.8%) clients had a record of receiving emotional support from a UCP. From the progress notes, the category *implementing interventions* included supportive interventions such as touch and listening.

*“Listening to music and verbal communication with me asking open and closed questions. Wanted me close and to hold her hand. Enjoys hand massage and holding hands and looking at family photographs.” (Chart 53)*

Participants also confirmed the importance of emotionally supporting clients:

*“You are there to do your utmost best to give them the comfort, to allow them to pass in peace. To allow them to be comfortable even if it’s in like singing, humming or whatever, all of that. You know the person, after a while, you get to know what the person likes, whether it be reading a bible, reading some chapters. Give them that comfort level.” (UCP4)*

Participants viewed emotional support for the family as an integral part of caring for clients at the end of life.

*“Support, because some of them [families] just don’t know what to do. With the clients that I have had, talking to them and letting them know that you are there. They do know that you are there, but they are not responsive to you, and some family members find it very difficult to deal with stuff like that. So that when you are there, you are helping them [families]as well to acknowledge what is happening and giving them the support – a shoulder to cry on. That is what I have run into working with the palliative.”(UCP4)*

Providing information on the clients’ condition and advice were other ways participants comforted the family:

*“I think just to keep them [family] calm. I think that they [family] depend on us a lot. From experience, I find that they will ask you questions pertaining to that, “Is she sleeping well? Is she restless?” The more you are able to respond with positive answers, it seems to keep them calm. But if you give them negative reports, like bad sleep or restless, they get wound up.” (UCP2)*

Interestingly, the task list grouping *family support* in the charts, which combined UCP task list headings family communications, respite, resources/planning, information sharing, and support for grief and loss, accounted for only 1.5% (*n* = 208) of UCPs’ activities (Fig. 1).

### The workplace environment

The theme *“The workplace environment”* comprises three subthemes that represented the unique features of the context of care, including UCPs’ roles within the team, preparation for their roles and responsibilities, and the emotional aspects of the work of caring for older clients and their families at the end of life.

#### Part of the team

The team comprised of amongst others UCPs, regulated healthcare professionals (HCPs) such as physicians, pharmacists, nurses, physiotherapists, occupational therapists, and FCGs. As nurses conducted clients’ assessments and developed the plan of care, all clients received a nurse home visit. UCPs occasionally met with other UCPs and HCPs to be shown a procedure such as a delegated task, or for overlap to provide care. However, UCPs mainly worked alone or with FCGs. Instead, information sharing was through documentation in the CITH; in particular, the progress notes in the category *information-sharing.* Despite the lack of in-person interactions, participants described a strong sense of being part of a team.

*“Everything intertwines. You are a team; you can’t do it by yourself. If you have a client, that person, for instance, will have bed sores and the sore needs to be dressed. You have to communicate and let the nurse know that this person needs attention in this area. So, you are in every way 100% part of the team. In order to view a person and know that this person is not comfortable, because it is not my duty or job to administer medication. I can visually see that this person obviously is uncomfortable, has pain. All that communication trickles down to the nurse to help relieve the pain or the discomfort.” (UCP4)*

Participants understood how their role fitted within the team, and because of their familiarity with clients, they felt like a valued member of the team.

*“I don’t think we have to identify solely what the needs are; we have a team that does this assessment. I feel like I am not the only one to address the needs of clients and families, but we have to be part of the team in identifying this. Our voices are heard, and they value our opinions since we are there with the clients longer.” (UCP1)*

Ongoing communication was a vital part of feeling they were part of a team. If issues arose that were beyond their scope or required immediate attention, then participants contacted their supervisor, who was a nurse, by telephone or email.

UCPs’ frequently encountered FCGs in the home. On the whole, they worked collaboratively with FCGs to meet clients’ needs as (described in the subtheme *helping out*). Having FCGs present was seen as a benefit by UCPs as FCGs were knowledgeable about clients and were valuable for informing UCPs’ care, and assisting with care (e.g., lifts). Added to this, FCGs had more latitude than UCPs and could assist by administering medications (e.g., analgesics), which facilitated timely interventions. However, difficulties occurred when UCPs felt that FCGs actions did not reflect clients’ wishes or clients’ best interests. Occasionally, clients and families misunderstood UCPs’ role and expectations, as one participant described:

*“Sometimes when we go into the home, it can be a communication barrier with the client themselves where I can’t get them to understand or cooperate to what I am supposed to be there for, which would be for personal care.” (UCP6)*

#### Prepared for the role

The subtheme “*Prepared for the role”* encompassed not only the preparation of UCPs with the knowledge and competencies to perform their functions, but also the assignment of clients to UCPs and compatibility between the client and family’s needs, and UCP’s abilities. When asked about their competency in meeting the needs of older clients and families, participants expressed confidence in their abilities. All participants had received training in palliative care or were currently in the process of gaining certification. Participants viewed education and training through community colleges and organizations as a vital step in developing competencies. A strong emphasis on training by their organization meant that participants’ felt supported, with on-going opportunities to gain new knowledge and skills inside and outside of the organization.

*“Before I had no training, I did not know my client was going to die; it happened really fast. I was able to notice the breathing was different, but I didn’t think the client was going to die on my watch. He passed away so fast, and I didn’t expect this. With training – it made things easier; I could see certain patterns that were not the same; the process of the death is not the same for everybody”. (UCP1)*

Participants described some of the courses relevant to the care of older clients, such as care of clients with dementia, safety, and lifts/transfers. Having the necessary competencies to fulfill their roles came not just from formal education and training, but also experience and personal aptitude, and interest. Most participants had considerable knowledge and experience working as UCPs for many years. As UCPs’ supervision was at a distance, this meant that they had more autonomy than UCPs in in-patient settings where a HCP is on-site. Consequently, UCPs had to deal with issues as they arose. Changes in the client’s condition meant that UCPs had to adapt to changing situations. Care of clients was complicated. Analysis of the charts revealed some incompatibility between clients’ and families’ needs and UCPs’ competencies, which created challenges and delays. For example, when the UCP was not authorized to perform a task (e.g., delegated lift), an FCG was not available or able to assist the client (e.g., administer medications), or the UCP did not have the necessary competencies. As the following account illustrates:

*“Sometimes, you are asked to do certain things that you are not delegated to do. Sometimes it is a lift, and you are not delegated to use the lift, they have to do it because you do not have the authorization to use the lift. You need the go-ahead from your supervisor to do that. Everything needs to be delegated and make sure that you are able and capable of doing certain things. So, if it is not within my scope of work, you can’t, so therefore you have to say that you are unable to do that.” (UCP3)*

Incompatibility between what was needed and what the UCP could do presented challenges and delays in meeting clients’ needs.

*“Pain. We can't do much with as we can’t give medications. We can get the meds out or the family can. We had a client that could not press the pain pump so he would say “please can you push the pump.” As there is not a nurse around then, I have to contact the office to come over to sort out the pain.” (UCP10)*

The assignment of clients to UCPs was essential for ensuring that UCPs were equipped to perform their responsibilities. There were specific processes and procedures concerning the assignment of clients to participants. Using a computerized management system to facilitate the process, a care coordinator would assign clients based on their needs, UCPs’ knowledge (based on training courses), interest, availability, and the client’s location. The process gave UCPs some choice, and all participants felt that their organization was supportive of their client assignment. Reassignment of UCPs was also possible if issues arose, as one participant described:

*“A lot of the time if you are experienced with palliative, you will probably get the first choice with that. Work comes, and you see the age of the person, and you look at the criteria, what the workload is, and you feel you are capable of. You call the office and you say I will go to see that client, or it is in my area. I will take the client on as a fill-in or ongoing. So, yes you have a choice.” (UCP3)*

#### Emotional but meaningful work

All participants described how caring for clients who were at the end of life was meaningful to the clients, families, and them. Their interest in caring for these clients came from a deep satisfaction that made UCPs’ feel valued at being able to make the client’s and family’s journey as smooth as possible. At the same time, providing care to clients and families was emotionally challenging. Participants were aware of client-UCP boundaries, but building connections with clients and families meant that it was sometimes emotional for them.

*“It was very hard because I had a woman, she almost died in my arms, and it was hard. I was there from Monday to Saturday, and I felt like I was the one that she wanted to talk to. When I would shower her and wash her hair, she always had a smile on her face. She would tell me that I was the only one who would treat her like a human being. It was hard.”(UCP5)*

Knowing they were doing their best to support clients and families helped participants to cope. Their personal experiences dealing with death and dying, and beliefs were helpful in dealing with highly emotive situations. Participants also described the importance of having a supportive supervisor that they could contact if the situation became emotionally difficult. Having the option to take clients with life-limiting conditions was also helpful. One UCP described how she took time away from caring for clients who required palliative care.

*“They really depend a lot on you. A lot of moral support, it gets tear-jerking. It can get somewhat difficult at times, and with my agency, they do have things in place where when it gets to that level where it is so stressful. They have help where you can talk your emotional feelings out as to the situation that has occurred. You have that support outside of work within the agency.” (UCP4)*

Dealing with families’ emotional reactions was particularly difficult. Personal experience with losing a loved one helped participants appreciate families’ perspectives and actions. Participants were able to empathize and use their experiences to help direct their care.

*“There are challenges with the family where it is hard for them to let go, and I have had instances where they are just holding on until the very end. Hope is there, and you can’t kill that hope. You just have to let them accept it on their own terms. But you know that they are dying, and they are saying, “No, no.” They will try to keep them up and going. Hope is something that everybody holds on to, to the very end.” (UCP4)*

## Discussion

We aimed to identify the types and frequencies of tasks performed by UCPs, and describe their engagement in home-based palliative care to older clients with life-limiting illness and their families, as well as identify barriers and facilitators to their work. Research in this area is limited and has been criticized for its methodological quality [10]. The lack of research attention is somewhat surprising given the significant role UCPs have in the provision of direct client care, but to some extent, reflects a general undervaluing of the contributions of UCPs [24]. One of the strengths of our study was the rigorous mixed methods approach. The quantitive data provided information on the types and frequencies of UCPs activities; while the qualitative methods provided greater depth in understanding the broader scope of UCPs’ roles and their enactment in practice. Through the qualitative methods, we were able to explore the processes, interactions, and intangible aspects of UCPs involvement in palliative care, including barriers and facilitators to their work. By integrated our data through merging, we were able to build on the strengths of the quantitive and qualitative methods [23]. Our data integration and presentation of the findings as an integrated thematic structure provided insights into similarities and differences in the data from the methods, and a comprehensive understanding. Our results are discussed in relation to what is currently known and recommendations based on the findings are made throughout.

Our study indicates that UCPs have a significant role in the provision of direct client care and support for clients and families receiving palliative care in the home. Consistent with studies examining UCPs’ roles in palliative care in the home, we found that supporting clients with activities of daily living is a central role [12, 13]. The nature of UCPs’ tasks is integral to palliative care and has the potential to impact clients' and families’ quality of life [11]. Participants recognized that through the provision of care, they could make a difference by treating clients with respect, honouring clients’ and families’ preferences, and providing physical comfort through interventions. The care described by participants and documented in the progress notes of the charts revealed an approach to care that was client-centered, with a willingness by UCPs to forgo assigned tasks if necessary. Sims-Gould and Martin-Matthews suggest that “bending of the rules” may be an attempt by UCPs to personalize care [25].

A related finding was that participants took on tasks such as housework that went beyond the assigned task of cleaning after providing care. Participants indicated that housework was an indirect way of supporting clients and families. In their examination of strategies used by UCPs in the delivery of home care to older clients, Sims-Gould and Martin-Matthews found that UCPs are willing to assist in other ways by substituting one task for another [25]. The substituting of tasks reflects a desire by UCPs to meet clients’ and families’ needs but also highlights a need not identified in the tasks assigned. Organizational emphasis on direct client care may result in other tasks such as housekeeping are relinquished, leaving a gap in services. Without support for housekeeping, clients and families may be challenged to meet this need, or UCPs may juggle housework with direct client care, reducing the time they have available for client care. This finding is not unusual. Devlin and McIlfatrick in a survey of UCPs who provided palliative care in the community found that just under half the sample identified that they “sometimes” performed duties not assigned to them [13].

The personal nature of the care provided by UCPs and their continued presence brought them in close contact with clients and families. Familial-like expectations and bonds can develop between UCPs and older clients [26]. Many of the roles and responsibilities of UCPs in direct client care were previously the preserve of nurses [8]. Malone’s work on the nurse-client relationship has resonance here [27]. Of pertinence is what Malone describes as *physical proximity,* which involves the care of the body and touch that brings nurses into the world of those they care [27]. It is through this proximity that nurses come to know their clients and families, or *narrative proximity* [27]. Participants’ relationships with clients and families were essential for client-centred care. The proximity described by Malone is necessary for developing therapeutic relationships that take care beyond the physical domain and for advocating for clients and families, or *moral proximity* [27]. Similar to others, we found that through their connections, UCPs got to know clients and families to provide care social, emotional, and practical support [12, 13]. UCPs' roles are evolving with respect to discrete physical tasks and controlled acts. However, there is little understanding of their preparation to deal with broader psychosocial and existential issues experienced by clients with life-limiting illnesses and their families.

The relational and affective aspects of UCPs’ role were meaningful to participants. Through their engagement with clients and families, UCPs can derive a sense of satisfaction at being able to make a difference in their lives [13, 25]. The close bonds that UCPs develop with clients, however, can expose them to emotional distress and grief at the loss of a client for which they may not be prepared [28, 29]. Participants in our study described the emotional toll of palliative care. Colleagues are a source of support for UCPs in long-term care settings [29]; however, UCPs in the home setting tend to work in isolation and have little contact with other UCPs or HCPs. Emotional support from their supervisors and choice over client assignments helped participants in our study cope; reducing exposure to death and dying, and issues of over-attachment. To protect the health and well-being of UCPs working in the home, this type of organizational support is imperative [17].

The reduced proximity of HCPs to clients and their families is a concern as it limits opportunities for HCPs to know and comprehensively assess clients’ and families’ palliative care needs. Without proximity, there is greater reliance on UCPs because of their contact with clients and families. Indeed, UCPs are referred to as the *“eyes and ears”* of the home health system [30]. Participants in our study felt they were part of a team and valued as team members. Participants realized that the frequency of their interactions with clients and families, and through the intimate nature of their work that they were informative to the healthcare team. Interestingly, despite their knowledge of the client and family, participants were not involved in the plan of care; a finding observed by others [4]. A high proportion of clients in our study had multi-morbidity, functional impairments, and symptoms; thus, a central responsibility for UCPs was the monitoring of clients. Participants’ observations centred on clients’ physical status but also included their psychological status; revealing some appreciation of holism. Studies in the home and long-term care settings have found that checking clients’ condition and reporting observations to HCPs as a UCP responsibility [10]. However, it is important to recognize that UCPs’ reports are likely to be limited without an appreciation of the observations or multi-faceted nature of palliative care.

Although UCPs are most often the direct care provider in the home, their work is assigned and tasks delegated by an HCP (typically a nurse), who is responsible for coordinating and managing home care services [4, 8]. To delegate effectively, nurses must ensure compatibility between the needs of clients and families and the competencies of health care providers assigned to address their needs. However, delegation is compounded by a lack of standard education for UCPs, varying titles, and competencies [31]. A computerized management system was used by care coordinators in our study to facilitate the process of UCPs’ assignments, taking into account UCPs’ competencies and preferences. Participants described feeling comfortable caring for older clients with life-limiting illnesses and their families. Certainly, preparation in palliative care and gerontology was important, so too was on-call support from their supervisor to address any concerns. This finding supports studies investigating the benefits of education and training to develop UCPs’ competencies in palliative care, and the recommendation that UCPs receive appropriate training [11, 32]. Although we identified *controlled acts* within the interventions implemented by UCPs; like others, we found they comprised a small percentage of activities undertaken by UCPs [14].

Consistent with other studies, we found that family helped manage the care of the client by supporting UCPs and vice versa [33]. Moreover, FCGs’ performed tasks and initiated care that UCPs could not, which was crucial for timely intervention. Interestingly, the expectations placed on FCGs were greater than those of UCPs; yet preparation is often lacking [5]. Although family can be considered a member of the healthcare team, they are also recipients of care. In their interviews, participants described emotionally supporting families as part of their role, although, there was little evidence of this support in the charts. It is not known why this disparity exists. It is possible that an organizational emphasis on physical care means that charting these aspects of care takes precedence. Alternatively, UCPs are engaging in activities that are not assigned to them. Either way, the charts do not entirely reflect what is happening in practice. Our findings support others in showing that UCPs are taking on these affective roles [10]. UCPs proximity in the home means they are ideally positioned to monitor and support families. We recommend that emphasis be placed on families’ needs as part of a holistic approach to palliative care in the home, to pre-empt, identify, and attend to families’ ongoing needs. To take on this role, UCPs should receive adequate training and time within their workload.

The home setting is unique and presents opportunities for UCPs to develop relationships with clients and families in their homes, but also challenges [34]. UCPs had to know when and how to respond to any given situation and adapt to a changing environment as oversight is at a distance [16]. Even with training and experience, and the efforts of the organizations to ensure compatibility between clients’ needs and participants’ competencies, the unpredictable and changing nature of clients’ conditions resulted in what has been described as *crises* [34]. Crises encountered by UCPs in the home are unpredictable events that occur during the provision of care that can adversely affect clients, families, and UCPs, and are not uncommon [34]. Crisis in our study occurred most often because of the change in clients’ condition and symptoms, UCPs made efforts to alleviate symptoms or notified their supervisor but were limited in what they could do and in some instances had to rely on FCGs or HCPs. Reliance on others to meet clients’ needs can potentially delay interventions and adversely affect not only the client experiencing the event, but the family and UCP witnessing it. The findings raise questions regarding the delegation of discrete tasks such as hygiene and grooming that are within UCPs’ competencies to clients with multifaceted care needs. Although older clients’ conditions and symptoms are to some extent unpredictable, they are anticipated given the often complex needs of older clients with a life-limiting illness. We observed an increase in home visits from HCPs leading up to the clients’ death or discharge; possibly as a result of an increase in the complexity of care.

Though infrequent, other crises in our study were *relational* and *organizational* in origin [34]. Relational crisis occurred when there were negative interactions between clients, families, and UCPs. Verbal aggression from clients, and refusal to cooperate with care occurred. Also, differences between UCPs and families approach to client care. A significant observation was racism and prejudice toward two UCPs from clients and families; a finding not uncommon in the literature investigating UCPs’ work conditions [34]. In comparison to the general working population, UCPs comprise a higher proportion of people who are visible minorities and have immigrant status [6]. Participants in our study reflected this diversity. Organizational crises stem from the disparity between organizational practices and operations and those providing or receiving the services [34]. Participants in our study had a clear understanding of their role in relation to other team members and what was expected of them. However, clients’ and families’ on occasion misunderstood the UCP role. Also, families’ expectations of having a nurse provide care rather than a UCP occurred in two charts. These crises can be a source of tension and dissatisfaction for clients, families, and UCPs [33, 34]. In preventing and managing crises, we recommend that there are clear expectations regarding UCPs’ roles, compatibility between the client’s and family’s needs and those of the UCP assigned, and policies on what constitutes acceptable and respectable behaviour. Further, UCPs should receive training on anticipating and managing crises, as well as access to resources to support them in their role [34].

## Limitations

We acknowledge that there are limitations to our study. We only have the perspectives of UCPs, and not those of clients, families or other health care team members, so it is not possible to say how the care provided was delivered or received beyond what was reported. Our study was conducted in one LHIN and across three home care agencies. The culture in these organizations is supportive, and the value placed on UCP education and training. We recognize the study findings reflect the positive organizational culture. Further, our study is embedded within the broader social-political context of Canadian health care, which may limit its transferability. That said, it important to note that within Canada and across many developed countries escalating health care costs, cost-containment, and the increased use of UCPs to provide direct client care is a reality [8] As palliative home care is a preference for many clients, greater understanding on how best to serve the needs of clients and families within the current fiscal climate and support UCP in their role is essential. Our findings provide insights into what UCPs found helpful that could apply beyond the context of the study. For example, some level of choice over clients that require palliative care, continuity in client assignments, emotional and practical support for UCPs from supervisors, and importantly, organizational support for training and education. The reciprocal processes of communication and collaboration described in our study not only helped participants feel valued and supported as team members but as Dahlke et al. point out, is essential for effective teamwork [35].

Another limitation of our study was the CITH. When using retrospective chart reviews the quality of the data is affected by what is available. We found that the UCP task list was restricted and provided little information beyond whether a task was completed or not. For this reason, we collected information from the progress notes to expand on UCPs' roles and activities. Based on our observations, we would encourage the use of progress notes to fully capture clients’ and families’ care including interactions, observations, monitoring, and care that cannot be transmitted through the use of box to indicate completion. Moreover, the documentation of care through the progress notes is more fitting with a palliative care approach, where the emphasis is on individualized and holistic care rather than a task-orientated approach. Notably, documentation by UCPs in the progress notes varied considerably and was sometimes missing or vague. It is also possible that some types of information were not recorded. For example, in their interviews UCPs discussed the importance of family support; yet data from the charts revealed that family support accounted for only a small proportion of UCPs’ activities (1.5%). Also, in 14 charts there was no indication of emotional support. As the charts are an essential part of communicating client care in the home, we recommend education and training of UCPs in charting care.

## Conclusions

The evolving home care landscape and demand for palliative care are challenging Governments to meet the needs of a growing older population. Consequently, there is an emphasis on cost-containment, and reliance on unpaid care from family caregivers, and services of UCPs. We found that UCPs provide palliative care with a focus on being genuinely supportive to older clients and their families. Their contributions to palliative home-care and the healthcare team are valuable. Through their presence, UCPs’ engage with clients and families and can develop bonds that facilitate a client-centred approach to care. UCPs' education and training are imperative to meet the increasingly complex needs of older clients and their families. Even with competencies in the provision of care, UCPs’ knowledge-base is limited. As UCPs’ roles evolve, care must be taken to ensure that UCPs are not expected to absorb the gap in care left by HCPs. Otherwise, there is a danger that clients’ and families’ needs may go unrecognized and unmet, and UCPs exposed to adverse working environments.

## Data Availability

Research ethics approval and permission to collect data from the organizations where recruitment occurred limits access to the raw data to the investigators (article authors). Therefore the raw data cannot be shared. A more detailed summary of quantitative data from the chart reviews can be provided by the first author (Dr. C. J. McPherson) upon request if the value of sharing is made explicit, and use of the material commensurate with the purpose of collecting the data. A summary of qualitative data is not available as the sample size and level of detail contained in the progress notes and transcribed interviews, may compromise clients’ and participants’ anonymity.

## Abbreviations

CITH: Chart-in-the-home
FCG: Family caregiver
HCP: Health care professional
ICC: Intraclass correlation coefficient
LHIN: Local Health Integration Network (LHIN)
UCP: Unregulated care provider
